# Accelerating snail vector genomics

**DOI:** 10.1186/s40249-024-01199-z

**Published:** 2024-05-06

**Authors:** Tom Pennance, David Rollinson

**Affiliations:** 1 College of Osteopathic Medicine of the Pacific – Northwest, Western University of Health Sciences, Lebanon, OR USA; 2 Global Schistosomiasis Alliance, Ealing Cross, 85 Uxbridge Road, Ealing, London, W5 5BW UK; 3 Science Department, Natural History Museum, Cromwell Road, London, SW7 5BD UK

**Keywords:** *Oncomelania*, *Bulinus*, *Biomphalaria*, Snail vectors, Genomics

## Abstract

The three most important genera of snails for the transmission of schistosomes are *Bulinus*, *Biomphalaria* and *Oncomelania*. Each of these genera, found in two distantly related families, includes species that act as the intermediate host for one of the three most widespread schistosome species infecting humans, *Schistosoma haematobium, S. mansoni* and *S. japonicum*, respectively. An important step in the fight against schistosomiasis in Asia has been taken with the publication of the article “Chromosome-level genome assembly of *Oncomelania hupensis*: the intermediate snail host of *Schistosoma japonicum*”, which means that genomes for all three major genera, including species across three continents, are now available in the public domain. This includes the first genomes of African snail vectors, namely *Biomphalaria sudanica*, *Bi. pfeifferi* and *Bulinus truncatus*, as well as high-quality chromosome level assemblies for South American *Bi. glabrata*. Most importantly, the wealth of new genomic and transcriptomic data is helping to establish the specific molecular mechanisms that underly compatibility between snails and their schistosomes, which although diverse and complex, may help to identify potential targets dictating host parasite interactions that can be utilised in future transmission control strategies. This new work on *Oncomelania hupensis* and indeed studies on other snail vectors, which provide deep insights into the genome, will stimulate research that may well lead to new and much needed control interventions.

**Graphical Abstract:**

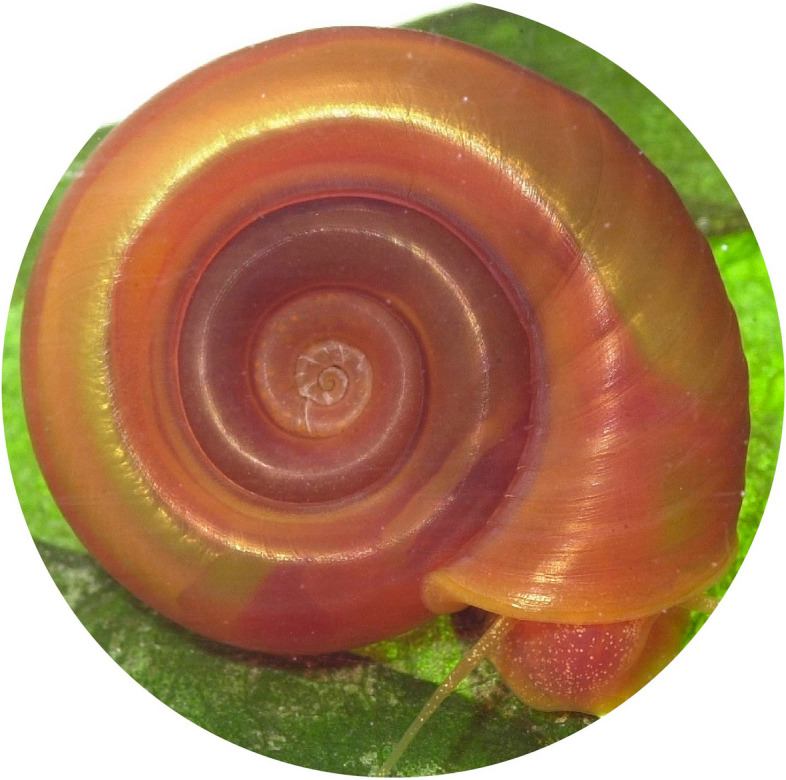

## Background

All countries are now tasked by the WHO 2030 Roadmap to achieve elimination of schistosomiasis as a public health problem, with some countries aiming to achieve interruption of disease transmission. The importance of an integrated approach to the control of schistosomiasis has long been recognised, and snail control is considered a key component of these strategies, alongside preventive chemotherapy [1]. Successes of such campaigns have been most evident in Asia, where interruption of disease transmission has been widely achieved thanks to the help of snail-focussed interventions, either removing the snail hosts or modifying snail habitats in endemic regions. The rationale is simple, if susceptible snails are not present in a habitat, there can be no schistosomiasis transmission. The practical problems have long been that environmentally friendly methods for snail control to use in all transmission settings are lacking, and that tailored interventions may often be unaffordable or impossible to implement. The difficulties for implementation of control are seen especially in some of the large freshwater bodies, such as the great lakes in East Africa, where simple chemical mollusciciding remains ineffective and other environmental modification may have large undesirable knock-on effects. The analysis of genomic and transcriptomic information from snail vectors, along with pioneering methods to manipulate gene expression, offer exciting avenues for research regarding these disease vectors and future approaches for curtailing transmission of schistosomiasis.

### *Oncomelania hupensis* genome

*Oncomelania hupensis* is responsible for the transmission of *Schistosoma japonicum* in China, Indonesia, and the Philippines. This dioecious and amphibious snail species is the essential link in the life cycle of the schistosome parasite and is a target for control interventions. The genome of *Oncomelania hupensis,* recently published in *Infectious Disease of Poverty* [2], not only provides the first reference for this important freshwater snail vector, but also represents the first to be sequenced from the Family Pomatiopsidae. The relatively complete (BUSCO 91%) *O. hupensis* genome was sequenced using long and short read DNA and RNA sequencing methods. The final assembly of 1.35 Gb represents a genome larger than that of the Planorbidae snail vectors currently assembled (Table 1) and across one less chromosome (*n* = 17 vs. 18). Following annotation of the *O. hupensis* genome, evolutionary analysis via comparison of orthologous protein sequences to other available molluscan lineages demonstrated that divergence from a common ancestor with the aquatic snails *Pomacea canaliculata* and *Bellamya purificata* occurred approximately 152.7–191.6 million years ago. Liu et al. identified a significant number of gene family expansions and contractions in *O. hupensis*, several of which are perhaps indicative of the adaptation/transition of *O. hupensis* to an amphibious lifestyle since this divergence. In addition, several genes that were found to be under positive selection in the *O. hupensis* genome as well as gene families under considerable expansion were associated with neuronal synapse development, protein–protein interactions, and the immune system, all of which could provide further insights into the biological functions of *O. hupensis* and its interactions with *S. japonicum*.

**Table 1:** Comparison of the genome assemblies and key features of snail vector species: *Oncomelania hupensis* [2], *Biomphalaria glabrata* iM line [3], *Biomphalaria pfeifferi* [4], *Biomphalaria sudanica* [5], *Biomphalaria straminea* [6] and *Bulinus truncatus* [7]

	*Oncomelania hupensis*	*Biomphalaria glabrata*	*Biomphalaria pfeifferi*	*Biomphalaria sudanica*	*Biomphalaria straminea*	*Bulinus truncatus*
Genome length (Gb)	1.449	0.871^a^	0.772^a^	0.94	1.005	1.222
Number of contigs/scaffolds	2178	255^b^	505	6728	84,585	523
Protein coding genes	30,604	35,015	31,894	23,598	40,218	26,292
Complete BUSCOs (%)	91%	96%	96%	94%	87%	96%
**Available genomes (NCBI)**	**1**	**9**	**1**	**1**	**1**	**1**

^a^The genome sizes have been independently measured based on fluorescence cell sorting intensity and yielded higher genome sizes for *B. glabrata* iM (1.090 Gb) and *B. pfeifferi* (0.913 Gb)

^b^By incorporating Omni-C read data, the 255 scaffolds of the *B. glabrata* iM line have been further scaffolded to generate a 0.84 Gb chromosome level assembly representing 18 chromosomes (2n = 36) for this species [8]

### Comparative ‘omics for snail vectors of schistosomiasis

The first genome description of *O. hupensis* comes at an exciting time in the wider field of snail vector ‘omics, where for the first time, highly complete genomes and transcriptomes for three major genera, *Bulinus* [7, 9, 10], *Biomphalaria* [3–6, 11] and *Oncomelania* [2] are now in the public domain (Table 1). The whole genome of the South American schistosome vector species *Biomphalaria glabrata*, which still represents the primary laboratory model species for investigating snail-schistosome interactions, was first to be sequenced [11]. The *Bi. glabrata* genome description allowed for further insights into the immune function and gene regulation that make this species a suitable snail host for *S. mansoni*, building from previous findings that had identified potential immune mechanisms, and even specific genes, dictating schistosome infection dynamics [12]. The availability of additional *Biomphalaria* genomes [4–6], and the resequencing of *Bi. glabrata* to produce the most recent chromosome-level genome assemblies [3, 8], has provided further avenues for performing comparative genomics, exploring schistosome resistance mechanisms across the genus. This includes differential gene expression studies [13, 14], genome wide association studies [15], quantitative trait loci mapping [3] and novel analyses to identify putative pathogen recognition receptors under balancing selection [5].

One group of invertebrate defence molecules that has received probably the most attention in relation to *Biomphalaria* defence against schistosomes are the variable immunoglobulin and lectin domain containing molecules, particularly the fibrinogen related proteins (FREPs), in addition to C-type lectin and galectin related proteins (CREPs and GREPs respectively) [16, 17]. Significant expansions have been observed in FREPs of African *Biomphalaria* species suggesting their importance for radiating across the African continent amidst selective pressure [4, 5], yet no FREPs are found in *Bulinus* [7, 9]. Considering the drastically different evolutionary history between the endemic African species of these two genera, namely that *Bulinus* likely originated in Africa whereas *Biomphalaria* was introduced from South America more recently within the last few million years [18, 19], it is perhaps not surprising that the biologically important FREPs in *Biomphalaria* may not occur in *Bulinus*, despite the presence of less complex precursor molecules containing fibrinogen domains.

Further analysis will determine whether *O. hupensis* mounts comparable, or drastically different, immune responses to schistosome infection as its *Biomphalaria* and *Bulinus* counterparts, as well as establish how much intraspecific diversity is evident given observed phenotypic differences in vectoral capacity (i.e. see discussion on smooth vs. ribbed shelled *Oncomelania* [2]). It will be fascinating to learn how different/similar the defence mechanisms are that have evolved in snail vectors that belong in such taxonomically separate snail families. Given the complexities of any animal immune system, it is perhaps unsurprising that a wide range of immune-related factors including pathogen-recognition receptor, non-self-recognition, signalling and effector molecules, have been associated with schistosome detection and elimination in *Biomphalaria* [12] and other trematode-gastropod systems [20]. The method used to establish these genes is nontrivial given their different functional roles, with some immune genes being characterised by conserved domain structures as a result of positive selection, whilst others are rapidly evolving under balancing selection due to the simultaneous arms races between the host and pathogens making their identification more difficult (see [5]). In the case of effectors and enzymes, gene expression profiles can be correlated to resistance profiles, yet allelic variation may dictate such differences [21]. All in all, this means multi-faceted analytical approaches need to be undertaken to tease out the mechanisms employed among snail vector species to combat schistosome infections to then establish the genetic factors that could inform future schistosome transmission control strategies.

### Further down the spiral

Detailed genomic and transcriptomic data will increase our understanding of the genetic diversity and population structure of *Oncomelania*, *Biomphalaria* and *Bulinus*. Although research across the three main genera of schistosome snail vectors is at different stages, the accessibility of next generation sequencing and the backbone of reference genomes from representative species of each genus will facilitate future studies. Whole genome analyses have the capability to resolve the many outstanding questions surrounding the evolutionary history, population diversity and functional responses of these gastropod vectors, as well as investigate the evolutionary arms race taking place with these parasites. Questions such as those poised by Liu et al. regarding the dynamics of schistosomiasis transmission in different regions of China; including the seemingly different vectoral capacity observed between *O. hupensis* with shells that are ribbed and non-ribbed [2].

Other complexities may be answered by population genomics, such as robustly interrogating and determining species boundaries between the proposed *Biomphalaria* morphospecies of Lake Victoria, *Bi. sudanica* and *Bi. choanomphala* [5, 22]. Determining species boundaries such as this is not only of evolutionary interest, but also allows for effectively targeting control to correct ecological environments and helping elucidate morphological differences that support a malacologists field identification of snails. This is true also for *Bulinus* species, such as those within the understudied *Bulinus africanus* and *Bu. forskalii* species groups which act as major hosts for multiple *S. haematobium* group species infecting humans and animals across Africa, investigation of which, promise to provide valuable insights into which factors contribute to unusual biological differences and compatibility within this diverse genus. Such biological differences include the environmental adaptations of some *Bulinus* species to withstand drought and desiccation, as well as colder climates in Mediterranean regions. Other genera such as *Neotricula* would also benefit from genomic investigations to clarify the taxonomic status of the different strains of *Neotricula aperta* and their role in the transmission of *Schistosoma mekongi* in Asia, a schistosome species which also recently had a chromosome-scale genome assembled [23].

An overarching goal is that snail vector omics will contribute towards reaching 2030 WHO goals for the elimination of schistosomiasis. So far, the research community has learnt a lot in terms of describing some of the fundamental immune mechanisms from the resources available for *Biomphalaria glabrata*. However, it is imperative to expand this research and data generation to encompass the other snail vector taxa. The next steps are of paramount importance, these being to validate functions and interactions of shortlisted genes and confirm how they influence phenotypes in these genera, most likely through differential expression analysis, gene knockdown, or gene over-expression studies [24, 25]. Findings from these functional studies could lead to novel approaches for controlling schistosome transmission, or directly controlling snail vectors themselves if factors concerning gastropod development, survival and environmental adaptation are uncovered.

## Conclusions

Complete genome assemblies, along with transcriptomic data, for the three major snail genera involved in the transmission of *Schistosoma* species: *Oncomelania*, *Biomphalaria* and *Bulinus*, mark a significant milestone in snail vector research. These comprehensive datasets offer exciting possibilities for exploring both the mechanisms governing host-parasite interactions through functional research and in clarifying the taxonomic boundaries between snail species complexes, which will aid in targeting and tailoring schistosomiasis control measures. To further capitalise on, and to begin translating the findings of omic studies into practical applications for disease control, open communication and collaborative efforts among researchers, control program managers, policymakers and funders, are essential.

## Data Availability

All data included in the commentary are from cited references and are publicly available.

## Abbreviations

FREPs: Fibrinogen related proteins
CREPs: C-type lectin-related proteins
GREP: Galectin-related protein
